# Discussions Between People With Dementia and Care Partners About End-of-Life Care Planning

**DOI:** 10.1093/geroni/igaa057.526

**Published:** 2020-12-16

**Authors:** Megan Shepherd-Banigan, Cassie Ford, Emmanuelle Belanger, Courtney Van Houtven

**Affiliations:** 1 Duke University School of Medicine, Durham, North Carolina, United States; 2 Duke School of Medicine, Durham, North Carolina, United States; 3 Brown University School of Public Health, Providence, Rhode Island, United States

## Abstract

Advanced care planning (ACP) leads to better end-of-life (EoL) care. Yet, some care-partners are unaware of the person with dementia’s (PwD) preferences. Care-partners play an important role in urging PwD to consider their EoL care wishes early in their disease course and to document those wishes. However, it is unknown whether discussions between care-partners and PwD are associated with documenting EoL care plans. We apply generalized linear models to baseline data from the CARE-IDEAS study which includes a sample of patients who received an amyloid PET scan and their care-partners (n=1,672). We examine the association between PwD report of having discussed EoL care with their care-partner and PwD report of having documented their plans through an advanced directive, a living will, or designating a health care proxy. PwD who reported speaking with their care-partners about EoL care were 10% (marginal probability (MP) 0.10; 95% CI: 0.8, 0.13) more likely to have documented their EoL care wishes. Furthermore, if PwD and care-partners agreed that they had discussed EoL care, PwD were 7% (MP 0.7; 95% CI 0.04, 0.10) more likely to report that they documented their EoL care plans. The positive association between communicating with care-partners about EoL care and having formal EoL care plans suggests that the ACP process could be a systematic approach to increase the care-partner’s knowledge of PwD EoL wishes. These results also suggest that increasing involvement of care partners in ACP may encourage patients to document their wishes at end of life.

